# International Federation of Clinical Chemistry (IFCC) Education Division, Expert Panel of Quantities and Units: *A Protocol for the Conversion of Clinical Laboratory data*

**DOI:** 10.1155/S1463924689000441

**Published:** 1989

**Authors:** H. P. Lehmann, H. G. J. Worth, O. Zinder

**Affiliations:** 1 Clinical Chemistry Department King's Mill Hospital Sutton-in-Ashfield Nottinghamshire NG17 4JL UK


Journal of Automatic Chemistry Vol. 11, No. 5 (September–October 1989), pp. 223–226

### International Federation of Clinical Chemistry (IFCC) Education Division†, Expert Panel of Quantities and Units‡: *A Protocol for the Conversion of Clinical Laboratory data*

**H. P. Lehmann (US), H. G. J. Worth (GB) and O. Zinder (IL)**

Debate on the use of SI units in clinical chemistry has continued for many years. Despite the recommendations made by the International Union of Pure and Applied Chemistry (IUPAC) and the International Federation of Clinical Chemistry (IFCC) in 1967 [1] and 1979 [2], and the World Health Organization (WHO) in 1977 [3], to use molar units to report clinical laboratory data, many countries have not yet made a commitment to do this. However, the situation may change in the near future: Australia, Scandinavia and the United Kingdom have been largely committed to the recommended SI units, with a variable commitment in the remainder of Europe, ranging from virtually 100% in Switzerland to 20% in Italy and 5% in Germany. Canada converted in 1982 and the USA plans to do so in the future [4]. The manufacture and marketing of equipment and reagents is largely directed at these countries, which will make it increasingly difficult for those who have not changed, like most of Latin America and Asia, to communicate on scientific matters relating to their patients.

For these reasons, it is appropriate to reiterate the recommendations made by IUPAC and IFCC [2], to present some data on conversion factors and to offer guidance on procedures for conversion.

### Système International d'Unités

The Système International d'Unités (SI) was adopted by the General Conference of Weights and Measures (CGPM) in 1960 and was later accepted in principle by IUPAC and IFCC. The system defines seven base units which may be used singly, or in combinations, to describe most quantitative measurements. The combinations are the derived units which are algebraic expressions of the base units using mathematical operations. In addition, there are units in common usage which are not part of SI but are recognized by the CGPM because of their accepted universal usage, for instance the minute (min), hour (h), day (d), and litre (l). Of particular importance is the litre, which has been agreed as the fundamental unit of volume in clinical chemistry [1]. The designated symbol for the litre is l or L. The upper-case L has the advantage that it cannot be confused with the number one in typescript, and is now required for many journals. (In the UK and USA the use of L is an official recommendation.) For these reasons, L is used in this article. To permit a wide range of magnitude of quantitative measurement, the SI includes a set of prefixes to the units that is added to denote an increase or decrease in the order of magnitude of the quantity ranging from $10^{18}$ to $10^{-18}$. In clinical chemistry changes in orders of magnitude of $10^{2}$, are recommended [1], although prefixes for $10^{-2}$, $10^{-1}$, 10 and $10^{3}$ are an accepted part of SI.

### Arguments for and against the adoption of the mole

Mass units and units for amount of substance, the kilogram and the mole respectively, are both acceptable within the SI. In clinical chemistry use of the mole is preferred wherever possible, the main issues of the debate being:

*Arguments for:*

(1) The mole is scientifically appropriate, as most methods of analysis (spectroscopy, fluorimetry, immunoassay etc.) are based on the measurement of numbers of molecules and not their mass.

(2) The concentration of a calibration standard is defined unambiguously. It is unaffected by the chemical form of the material used, for example glucose standards may be prepared from anhydrous glucose or glucose monohydrate. A given volume of a standard solution, having a concentration of 10

Correspondence should be addressed to: H. G. J. Worth, Clinical Chemistry Department, King's Mill Hospital, Sutton-in-Ashfield, Nottinghamshire NG17 4JL, UK.

† Education Division members are: O. Zinder (IL), Chairman: H. G. J. Worth (GB), Secretary: C. Fraser (GB); N. de Cediel (CO); and A. Deom (CH).

‡ Expert Panel members are: H. P. Lehmann (US), Chairman: D. R. Bangham (GB); G. Ferard (FR); J. G. Hill (CA); M. Lauritzen (DK); H. Olesen (DK); and J. C. Rigg (NL).

© 1989 IFCC.

mmol/L, contains an unambiguous quantity of $C_6H_{12}O_6$, whereas a standard of 1·80 g/L (or 180 mg/dL) contains differing amounts of $C_6H_{12}O_6$, depending on which form of glucose is used in its preparation. This is a particular problem with therapeutic drug measurements where different salts and conjugates of a drug, perhaps with different degrees of hydration, are available and may be suitable both for administration to patients and calibration of analyses. Although pharmaceutical preparations are usually clearly marked in this respect, there is the possibility of confusion among medical and paramedical staff who do not fully appreciate that the same mass of different forms of the same compound will probably contain different amounts of the active component.

(3) Physiological relationships between substances commonly occur on a mole-to-mole basis, for example 1 mole of haemoglobin and 1 mole of $O_2$ yield 1 mole of oxyhaemoglobin. This is particularly important with pharmacological agents where active metabolites may be present, or different metabolites in the same metabolic pathway are being measured, for example lactate and pyruvate. Indeed, the use of molar units may help in understanding the molecular mechanisms of disease processes more easily.

(4) At the present time, many different units are used to express the concentration of some analytes (for example mmol/L, meq/L, mg/L, mg/dL for plasma calcium), which can lead to misunderstanding and misinterpretation.

(5) As more countries adopt the recommended SI units, it is going to become more difficult for the remaining countries not to do so. Increasingly, manufacturers and journals are going to use these units or require them to be used.

(6) There is, therefore, a strong argument for uniformity. This is particularly true in the field of therapeutic drug monitoring, where, for example, up to seven different units have been reported for measuring digoxin [5]. The same problem arises with many hormone assays, such as thyroxine [6].

*Arguments against:*

(1) The change to the mole has not been universally popular with clinicians in those countries where reporting in molar units has been implemented. Some do not see the need for change and are concerned about the risks of clinical misinterpretation as a result.

(2) In clinical chemistry laboratories balances are used to measure the quantity of materials for standards and calibrants in mass units. Consequently, there are risks that solutions may be prepared incorrectly if molar units are used instead.

(3) The reference and/or pathological range for some analytes are cumbersome, for example urate is expressed to three figures (μmol/L), or to three places of decimals (mmol/L), calcium to two places of decimals (mmol/L).

(4) Substances which are administered to patients and are subsequently measured in plasma, serum etc, are prescribed in mass units and should therefore be measured in the same units.

*Conclusions*

The strongest argument against the use of the mole is the one which suggests that confusion will be caused by the change. This is an argument against change and not SI units *per se*. If there was uniformity, this would be the strongest argument of all, but as there is not, the strongest argument for change is the one towards uniformity, to avoid the risk of confusion and misunderstanding that exists at present. If the need for uniformity is accepted, then the mole is the most scientifically acceptable unit.

Against this, the other points of the argument are insignificant. If some clinical chemists are unable to prepare solutions in molar concentrations correctly, their ability to prepare solutions in mass concentrations must also be questioned. Some measurements may seem to be cumbersome in molecular units, but, equally, so are some in mass units. This will cease to be a problem as molecular units become more familiar. There is no need to have cumbersome ranges for analytes, since the number of significant figures in SI units should be comparable with the number in traditional units and reporting increments should be selected so that no greater precision is implied by the use of SI units. Finally, the inter- and intra-patient relationship between the drug administered and measured in the patient's fluids is so variable that the need to express these in the same unit has little or no relevance.

### A protocol for conversion to the mole

It must be accepted that the conversion, from one set of units to another, may produce problems for both clinicians and laboratory staff, with the risk of misunderstanding and misinterpretation. The following protocol is suggested in order to avoid, or at least minimize, difficulties in the conversion:

(1) The complete programme should be detailed in advance, and must include a timetable with dates indicating when each step has to be completed. National, regional and/or local organizing committees should be appointed to co-ordinate this programme.

(2) Sufficient time must be allowed for each stage to ensure that the timetable is maintained, and staff are adequately prepared.

(3) An agreed changeover date must be decided at the outset and adhered to. This date should be agreed nationally, if possible, in order that all laboratories change at the same time. If this is not practicable, then regions or areas, such as cities and districts, should aim to change on the same day.

(4) The change on the specified day must be absolute. There should not be a 'run in' period during which the old and the new systems are used concurrently. *Laboratory staff should not offer to convert data back to the old units*, but should be able to assist in the intrepretation of data presented in the new system.

*Timetable for conversion programme*

It is not possible to specify exactly how much time should be allowed for the total conversion programme, as this will vary from country to country. However, time must be allowed to set up national advisory bodies, including a steering committee, as well as the local laboratory programme. It is difficult to see how all of this can be achieved in less than two years. The laboratory's preparation for conversion must include the following stages, to which suggested time periods have been assigned. Many parts of this programme need not (indeed, should not) run consecutively, as they can be achieved simultaneously.

(1) Agreement should be reached at an early stage on which quantities will be changed. This list must be comprehensive so that additional changes at a later date will be kept to a minimum. Guidance, if not complete authority for this should come from the national steering group. (Three months.)
(2) Development and implementation of an education and training programme for laboratory staff to ensure that they understand the purpose of the change, and that they are able to make the appropriate conversions, and advise clinical staff. (Six months.)
(3) Development and implementation of an education and training programme for other health-care personnel, for example nurses and pharmacists. This should include seminars conducted by members of the laboratory staff who can explain the purpose of the change and respond to questions. (Six months.)
(4) Charts and/or tables of conversion data must be prepared for all analytes, and be circulated to all health-care personnel. (Three months.)
(5) Redesigning and ordering of laboratory stationery etc., and conversion of analysers to take into account new units and reference intervals. (Nine months.)

It is highly desirable that the national steering committee should produce a document or booklet giving guidance on these points.

### Conversion data

The following list of the more frequently assayed serum or plasma components shows the conversion factors for the commonly used unit(s) to the recommended. (This is usually a conversion from mass to molar units, but not always.) Additional and more comprehensive listings can be found elsewhere [7–9].

This list, and the subsequent discussion, includes all those clinical chemical analyses for which there are WHO-recommended methods [10].

*Proteins*

Specific protein concentrations should be expressed in molar units where the relative molecular mass is known. This may also be preferred for albumin, when its concentration is being compared with other specific proteins or compounds bound to it, such as bilirubin. A relative molecular mass for albumin of 67 000 has been used to derive the conversion factor in the table. Serum total protein concentration is expressed in g/L, as no relative molecular mass can be assigned to a heterogeneous mixture of macromolecules. Albumin concentration may also be expressed in g/L in order to compare its concentration with that of total protein.

*Enzymes*

The recommended unit for the measurement of catalytic activity of enzymes is the katal (kat) [2], which is defined as the activity that will convert 1 mole of substrate per second under defined conditions. However, in most countries the International Unit (U) is still used, and it is defined as the activity which will convert 1 micromole of substrate per minute under defined conditions.

*Hydrogen ion*

Traditionally, hydrogen ion measurement has been made in pH units. In the recommended units this would be changed to nmol/L, the relationship between pH and hydrogen concentration being:

$$\mathrm{pH} = -\log_{10}[\mathrm{H}^+] \text{ where } [\mathrm{H}^+] \text{ is expressed in mol/L.}$$

A pathological blood pH range of 7·8 to 6·8 corresponds to a hydrogen ion concentration range of 16 to 160 nmol/L. The use of molecular units clearly demonstrates the wide range *in vivo*; this being greater than most of the commonly measured analytes in whole blood, plasma or serum. However, the pH scale, which is consistent with the SI, will continue to be widely used.

*Partial pressure of gases*

It is recommended that whole blood partial pressure measurements (i.e. $pO_2$ and $pCO_2$) should be reported in kilopascals (kPa), rather than millimetres of mercury (mmHg).

### Acknowledgements

The authors wish to acknowledge the helpful comments and suggestions made by the members of the IFCC Education Committee, the Expert Panel on Quantities and Units, and the IUPAC Commissions VII.2 and VII.3 and many other colleagues, particularly Professor L. F. Bertello, Dr A. Deom and Dr D. S. Young.

*Conversion factors for the more frequently assayed serum or plasma components.*

| Analyte | Old units | Conversion (multiplication) factor (old recommended) | Recommended unit |
|---|---|---|---|
| Acetaminophen (Paracetamol) | mg/L | 0·00661 | mmol/L |
| | mg/dL | 0·0661 | mmol/L |
| Albumin† | g/dL | 10 | g/L |
| | | 149 | μmol/L |
| Bicarbonate | meq/L | 1 | mmol/L |
| Bilirubin | mg/dL | 17·1 | μmol/L |
| Calcium | meq/L | 0·5 | mmol/L |
| | mg/dL | 0·25 | mmol/L |
| Chloride | meq/L | 1 | mmol/L |
| Cholesterol | mg/dL | 0·0259 | mmol/L |
| Cortisol | μg/dL | 27·6 | nmol/L |
| Creatinine | mg/dL | 88·4 | μmol/L |
| Digoxin | ng/mL, μg/L | 1·28 | nmol/L |
| | μg/dL | 12·8 | nmol/L |
| Glucose | mg/dL | 0·0555 | mmol/L |
| Iron | μg/dL | 0·179 | μmol/L |
| Lithium | meq/L | 1 | mmol/L |
| Magnesium | meq/L | 0·5 | mmol/L |
| | mg/dL | 0·411 | mmol/L |
| Phosphate | mg/dL | 0·323 (as phosphorus) | mmol/L |
| Potassium | meq/L | 1 | mmol/L |
| Salicylate | mg/L | 0·00724 (as salicylic acid) | mmol/L |
| | mg/dL | 0·0724 (as salicylic acid) | mmol/L |
| Sodium | meq/L | 1 | mmol/L |
| Thyroxine | μg/dL | 12·9 | nmol/L |
| Total protein | g/dL | 10 | g/L |
| Triglyceride | mg/dL | 0·0124 (as tripalmitin) | mmol/L |
| | mg/dL | 0·0113 (as triolein) | mmol/L |
| Urate | mg/dL | 59·5 (as uric acid) | μmol/L |
| | | 0·0594 (as uric acid) | mmol/L |
| Urea (Carbamide) | mg/dL | 0·167 | mmol/L |
| | mg/dL | 0·357 (as blood urea nitrogen) | mmol/L |

† See text for explanation of two recommended units being given for albumin.

### References

1. Dybkaer, R. and Jorgensen, K., *Quantities and Units in Clinical Chemistry Including Recommendation 1966 of the Commission of Clinical Chemistry of The International Union of Pure and Applied Chemistry and of the International Federation for Clinical Chemsitry* (Williams and Wilkins Co., 1967).
2. Dybkaer, R. *Clinica Chemica Acta*, **96** (1979), 157.
3. *The SI for the Health Professions* (World Health Organization, Geneva, 1977).
4. SI Units for Clinical Laboratory Data, *Journal of the American Medical Association*, **253** (1985), 2553.
5. Simpson, D., *Annals of Clinical Biochemistry*, **17** (1980), 328.
6. McLaughlan, D. M., Raggatt, P. R. and Zilva, J. F., *Annals of Clinical Biochemistry*, **12** (1975), 1.
7. Bold, A. M. and Wilding, P. *Clinical Chemistry Conversion Scales for SI Units and Adult Normal Reference Values* (Blackwell Scientific Publications, Oxford, 1975).
8. McQueen, M. J., SI Units. *A Practical Guide for Health Professions* (Simole Consultants Ltd, Ontario, 1982).
9. Baron, D. N., SI units. *British Medical Journal*, **4** (1974), 509.
10. Cediel, N. de, Deom, A., Hill, P. G., Sarkar, A. K., Vazquez, D. A., Olazabal, R. and Lothe, F., *Methods Recommended for Essential Clinical Chemical and Haemotological Tests for Intermediate Hospital Laboratories* (World Health Organization, Geneva, 1986).

*In addition the following bibliography in Spanish is recommended, compiled by Professor L. F. Bertello:*

Bertello, L. F., *Acta. Bioq. Clin. Latinoamer.*, **13** (1960), 3.

*Oficina Sanitaria Panamericana. Adopocion del S.I. Boletin OPs* (1978).

*Sociedad espanola de Quimica Clinica. Denominaciones, unidades y factores de conversion en Quimica Clinica* (1977).

Bertello, L. F. El S.I. *Su aplicacion en el area de la Salud* (EUDEBA, Buenos Aires, 1980).

Gomez Vesga, C., *Sistema Internacional de Unidades* (FECODEL, Bogota, 1980).

*OMS El SI paralasprofesiones dela Salud* (Ginebra, 1980).

Baez, A. de, *et al.*, *Sistema Internacional de Unidades. Fundamentos y aplicacion* (UASD, Santo Dimingo, 1983).

Bertello, L. F., *Rev. Asoc. Med. Argentina*, **96** (1983), 122–126.

Bertello, L. F., Abreviaturas normalizadas para Sistemas y Magnitudes. *Acta. Bioq. Clin. Latinoamer.*, **17** (1983), 465–469.

COLABIOCL, *Recomendacion 1986—Abreviaturas de Sistemas y Magnitudes.*

